# Conversion of Waste Cooking Oil to Rhamnolipid by a Newly Oleophylic *Pseudomonas aeruginosa* WO2

**DOI:** 10.3390/ijerph19031700

**Published:** 2022-02-01

**Authors:** Shu Shi, Zedong Teng, Jianwei Liu, Tinggang Li

**Affiliations:** 1 Key Laboratory of Urban Stormwater System and Water Environment, Ministry of Education, Beijing University of Civil Engineering and Architecture, Beijing 100044, China; 15901031225@126.com; 2 Innovation Academy for Green Manufacture, CAS Key Laboratory of Green Process and Engineering, Beijing Engineering Research Centre of Process Pollution Control, Institute of Process Engineering, Chinese Academy of Sciences, Beijing 100190, China; zdteng@ipe.ac.cn; 3 University of Chinese Academy of Sciences, Beijing 100049, China; 4 Ganjiang Innovation Academy, Jiangxi Province Key Laboratory of Cleaner Production of Rare Earths, Chinese Academy of Sciences, Ganzhou 341000, China

**Keywords:** waste cooking oil, *Pseudomonas aeruginosa*, rhamnolipid, salt tolerance

## Abstract

The components of waste cooking oil (WCO) are complex and contain toxic substances, which are difficult to treat biologically. *Pseudomonas aeruginosa* WO2 was isolated from oily sludge by an anaerobic enrichment–aerobic screening method, which could efficiently utilize WCO and produce rhamnolipid. The effects of nutrients and culture conditions on bacterial growth and lipase activity were investigated to optimize the fermentation of WCO. The results showed that strain WO2 utilized 92.25% of WCO and produced 3.03 g/L of rhamnolipid at 120 h. Compared with inorganic sources, the organic nitrogen source stabilized the pH of fermentation medium, improved lipase activity (up to 19.98 U/mL), and promoted the utilization of WCO. Furthermore, the WO2 strain exhibited inferior utilization ability of the soluble starch contained in food waste, but superior salt stress up to 60 g/L. These unique characteristics demonstrate the potential of *Pseudomonas aeruginosa* WO2 for the utilization of high-salinity oily organic waste or wastewater.

## 1. Introduction

Food waste (FW) is produced in large quantities by both catering industries and families. The total amount of FW produced rises rapidly with the expansion of population and economic development. FW is one of the most common urban organic solid wastes, and it was expected that by 2025, 2.5 × 10^9^ tons of FW will be produced worldwide [1]. In Europe and North America, 95–115 kg of FW was produced per person every year, and the total amount of FW produced exceeded one third of the total food production of those countries [2]. With a population of 1.4 billion, China produced a huge amount of FW with 125 million tons in 2020. The treatment of FW has become a key issue. FW in China was usually a mixture of various substances, including oil, vegetables, meat, rice, and noodles. Compared with western countries, China’s unique food culture and cooking habits promoted the consumption of edible oil, and then produced a large amount of waste cooking oil (WCO) [3]. Usually, WCO was mixed with FW and transported to the treatment facility. At a rough estimate, more than 90% of FW was mixed with municipal solid organic waste for landfill or incineration [4], and WCO decomposed in landfills and released large amounts of greenhouse gases. For biological facility treatment, the existence of WCO affected the entry of oxygen into the aerobic treatment system, and WCO also inhibited the microbial metabolic activity [5]. A previous study showed that 52.1% of families and 56% of catering industries in São Paulo discarded WCO in sinks, sewers, and drains [6]. According to statistics, the annual yield of the WCO is about 5 million tons in large and medium-sized cities in China [7]. Nowadays, the annual world consumption of edible oil was around 210 million tons, and the annual WCO production was estimated at 42 million tons [8]. The proper disposal of such a large amount of waste oil is a major challenge all over the world. The accumulation of WCO has caused potential environmental pollution risks, including water pollution, odorous gases, and pests [9,10]. Therefore, effective treatment strategies of WCO are urgently needed to reduce the harm of WCO to the environment.

Like other biomass such as microalgae or lignocellulose, WCO is considered one of the most abundant substrates for the transformation of valuable products [11,12]. On the utilization of WCO as a feedstock, the most well-known application is the production of biodiesel, which could reduce the utilization of petrochemical energy [13]. The European biodiesel market is known as the largest biodiesel market globally, and the third largest biofuel market in the world. In European countries, WCO is mainly managed by households and recycling departments, from which the recovery rates are 5.6% and 86%, respectively [14], and about 25% of biodiesel was produced from WCO. In the United States, the use of WCO as a raw material to produce biodiesel has received government subsidies, making the recovery rate of WCO close to 100% [15]. In China, the government provided various subsidies for WCO recyclers to encourage waste oil collection, but banned illegal WCO recycling so as to reduce the possibility of these waste oils returning to households after industrial treatment [16]. In addition, China’s energy development plan showed that 18 billion Chinese yuan were invested in biofuels (including bioethanol and biodiesel) in 2020 to encourage technological innovation and promote the production and utilization of biofuels [17]. However, the yield of biodiesel produced in China has decreased in recent years [7,15], which could be attributed to the high processing costs and the subsidy-dependent approach for biodiesel production and consumption in China.

Compared with other treatment methods, using microorganisms to treat WCO is one of the most environmentally friendly methods. Some bacteria and fungi can use WCO to produce biosurfactants. Fungi consumed more nutrients for their own growth than bacteria. On the basis of bioconversion of waste oil to value-added chemicals as a feedstock rather than biomineralization, biosurfactants produced by bacteria seem to have more practical significance. The biological treatment of WCO remains a challenge because the degradation of WCO requires a complex enzyme system, and the low solubility of long-chain fatty acids is a rate-limiting factor in the utilization efficiency [18]. Surfactants could reduce the interfacial tension, increase the bioavailability of oil substances, and promote the utilization of oil by microorganisms. Compared with chemical surfactants, biosurfactants have the advantages of high surface activity, biodegradability, and lower toxicity [19,20,21]. WCO contained fatty acid structural composition, so WCO could induce some strains to produce surfactants. According to (https://www.marketsandmarkets.com/PressReleases/biosurfactant.asp, accessed on 28 December 2021), the global biosurfactant market is expected to worth 5.52 billion in 2022.

Biosurfactants are broadly classified as glycolipids, lipoproteins or lipopeptides, phospholipids, fatty acids or natural lipids, polymeric surfactants, and particulate surfactants [22]. Biosurfactants, being secondary metabolites, are produced in the stationary phase of microbial growth. Based on their microbial origins, *Pseudomonas* species are known for their capacities to produce glycolipids. In previous studies, the surfactant produced by *Pseudomonas aeruginosa* was determined to be rhamnolipid [23,24]. *Pseudomonas aeruginosa* produced mono-rhamnolipid and di-rhamnolipid via the rhlAB operon that encodes the RhlA and RhlB rhamnosyltransferases [25]. Rhamnolipid has been widely used in various industrial applications with good biocompatibility, high surface activity, and tolerance to extreme environments [26]. Some researchers studied the production of biosurfactant rhamnolipid from palm fatty acid distillate [27]. A study reported the effect of adding glucose into waste frying oil culture medium to improve rhamnolipid production [28]. The production of rhamnolipid by *Pseudomonas aeruginosa* ATCC 9027 was positively correlated with the content of octadecanoic fatty acid [29]. At present, most of the reported carbon sources were frying oil and crude oil. WCO containing vegetable and animal oil was usually transported together with FW, leading to its deterioration and the production of toxins, such as phenylpyridine and aflatoxin [30]. As reported previously, some bacteria could not use WCO as a carbon source [31]. Thus, there is still a need to focus on efficient WCO utilization.

This study describes the characterization of a unique, oleophylic, halotolerant, wild-type *Pseudomonas aeruginosa* WO2. To avoid isolation of fungi, strain WO2 was isolated by the anaerobic enrichment–aerobic screening method, *Pseudomonas aeruginosa* WO2 can efficiently utilize WCO and accumulate rhamnolipid without additional nutrients. Although strain WO2 can use other common components in FW as nutrient sources, the strain exhibited inferior utilization ability of soluble starch but superior salt stress up to 6%. Strain WO2 is preferable to organic nutrient sources as it can be used for the treatment of high-salinity oily kitchen wastewater.

## 2. Materials and Methods

### 2.1. Sludge Samples and Culture Medium

Anaerobic medium (g/L): glucose 30, NH_4_Cl 0.3, yeast extract 1.3, NaNO_3_ 5, NaCl 1, MgCl_2_·6H_2_O 0.5, KH_2_PO_4_ 1, KCl 0.3, CaCl_2_·2H_2_O 0.015, and Na_2_S·9H_2_O 0.048. Fermentation salt medium (g/L): (NH_4_)_2_SO_4_ 5, KH_2_PO_4_ 2, K_2_HPO_4_ 2, NaCl 10, and MgSO_4_·7H_2_O 0.5. Nutrient broth (g/L): yeast extract 3, beef extract 5, and NaCl 5. The pHs of all the above mediums were adjusted to 7, using 6 M NaOH and 3 M HCl. After the pHs were adjusted, a fermentation medium was prepared by adding 10 g/L WCO to the fermentation salt medium. The medium was carried to be autoclaved at 121 °C for 30 min. Experiments were carried out in duplicates.

The WCO used in this experiment was collected from the FW trash can of the Institute of Process Engineering at different periods. After collection, the samples were mixed and sterilized at 121 °C for 30 min and stored at the laboratory. The commercial rapeseed oil used in this experiment mainly contained 6–14% linolenic acid and 55–70% oleic acid, which were purchased from the local supermarket. Other chemicals were analytical reagent grade and purchased from Kepujia chemical Company (Beijing, China).

### 2.2. Isolation and Identification of WCO-Utilizing Strains

In order to avoid the interference of fungi, the bacterial anaerobic enrichment–aerobic screening method was improved according to the method reported previously [32]. Briefly, in the anaerobic glove box, about 5 g of sludge sample was added to a 60 mL serum bottle containing 30 mL anaerobic medium. The serum bottle was sealed with a butyl stopper and aluminum cover to ensure that the interior was anaerobic. Then the bottle was incubated at 37 °C and 180 rpm for 7 days. Then, 5 mL of enriched culture was transferred to a fresh anaerobic medium. After two repetitions, 5 mL of enriched culture was transferred to the fermentation medium and cultivated at 37 °C and 180 rpm for 7 days. It was observed that the WCO in the serum bottle was consumed and the serum bottle producing surfactant was picked out. After dilution, the fermentation culture was spread on agar plates and incubated under the same culture conditions for 48 h. After the colonies with inconsistent morphology and color were selected, they were purified three times by re-streaking method, and then put into the fermentation medium again to test the WCO utilization ability and lipase activity. Finally, the strain WO2 with the superior WCO consumption performance was selected and sequenced by 16S rDNA. The 16S rDNA gene sequence of the isolated *Pseudomonas aeruginosa* WO2 was deposited in GenBank of NCBI under accession number SUB10857597, and the phylogenetic analysis was constructed by Neighbor-Joining method in Mega 7 software.

### 2.3. Optimization of WCO Consumption Conditions

Seed culture was prepared from nutrient broth medium and then transferred to fermentation medium with 4% (*v/v*) inoculation. In order to investigate the optimum conditions for the consumption of WCO, the carbon source (rapeseed oil, WCO, glucose, and soluble starch), nitrogen source ((NH_4_)_2_SO_4_, NH_4_Cl, NaNO_3_, yeast extract, and peptone), inoculation ratio (2–10% *v/v*), temperature (30–40 °C), and pH (5–9) were performed. After fermentation for 24 h, the optical density and lipase activity in the broth were measured. All the optimization experiments were conducted in a 100 mL serum bottle containing 50 mL fermentation medium.

Under the optimized conditions, the fermentation was carried out for 5 days to evaluate the best consumption effect of strain WO2 feeding WCO. The pH, lipase activity, oil utilization rate, and optical density were assayed every 12 h during the fermentation. Rhamnolipid were determined by a spectrophotometer (N5000, YouKe, Shanghai, China) every 24 h.

### 2.4. WCO Tolerance Test

*Pseudomonas aeruginosa* WO2 was inoculated into the fermentation medium under optimal conditions and cultured at 37 °C and 180 rpm. As the sole carbon source in the fermentation medium, the concentration of WCO was changed to 10, 20, and 40 g/L. The samples were collected after 120 h to determine the oil consumption rate and rhamnolipid production.

### 2.5. Salt Stress Test

To investigate the effect of salt contained in FW on the growth of strain WO2, the concentration of salt (NaCl) in the fermentation medium was changed to 10, 20, 40, 60, 80, and 100 g/L. *Pseudomonas aeruginosa* WO2 was transferred to the fermentation medium with optimal parameters and cultured according to the fermentation conditions. The non-inoculated fermentation salt medium was used as a control, and the OD_600_ value in the medium was measured at 24 h. The tolerance to bacterial culture was determined by optical density.

### 2.6. Cell Density and Enzymatic Assay

Cell density was measured using a spectrophotometer (N5000, YouKe, China), at 600 nm after appropriate dilution.

For the lipase activity assay, 0.5 mL of cultures were harvested in each period. The lipase activity was determined using cell-free supernatant. Briefly, lipase activity was assayed by measuring the amount of p-nitrophenol released from 4-Nitrophenol palmitate (p-NPP) [33]. Next, 0.24 mL of p-NPP solution (A) and 2.16 mL of 0.05 M Tris-HCl (pH = 8) (B) were treated with 0.1 mL culture supernatant and incubated at 37°C for 15 min, then 2 mL ethanol was added to terminate the lipase activity test. The optical value was measured at 405 nm (OD_405_). One unit (U) of lipase activity was defined as the amount of lipase that release of 1 μmol of p-nitrophenol per minute from p-NPP.

The formula of solution is:

A: 300 mg p-NPP + 100 mL isopropanol + 25 mL Triton-X-100. B: 0.05 M Tris-HCl (pH = 8). A: B = 1:9.(1)

### 2.7. WCO Consumption

WCO in the medium was gradually consumed. In order to determine the residual oil content in the fermentation medium, the residual oil in the culture medium was extracted and separated with n-hexane. The operation of this experiment adopted the method reported in a previous study [34]. We added 25 mL n-hexane to the serum bottle and mixed it vigorously for 5 min. We separated the upper n-hexane phase with a separating funnel, and then collected the remaining WCO with a rotary evaporator (N-1300, EYELA, Tokyo, Japan) and weighed it.

The WCO utilization rate was calculated by the following formula:

WCO utilization rate% = (m_initial oil_ − m_residue oil_/m_initial oil)_ × 100% m_residue oil_: Amount of WCO remaining in the culture medium after microbial metabolism. m_initial oil_: Amount of WCO initially added to the culture medium. (2)

### 2.8. Rhamnolipid Production

The concentration of rhamnolipid was determined by sulfuric acid-anthrone colorimetry. Anthrone could complex with the rhamnose motifs in the rhamnolipid structure, color reaction occurred under sulfuric acid conditions, and the rhamnolipid content could be calculated according to the concentration relationship between rhamnose and rhamnolipid [35,36]. Firstly, 1.5 mL of fermentation fluid was centrifuged at 12,000× *g* rpm for 10 min to remove cells and residual WCO. After the fermentation sample was diluted, sulfuric acid-anthrone solution was added for full reaction. The sample’s absorbance was determined at 625 nm. According to the rhamnose standard curve (0–100 mg/L), the rhamnose content in the fermentation sample was determined. In the preparation of sulfuric acid-anthrone solution, we dissolved 0.02 g anthrone in 10 mL concentrated sulfuric acid.

## 3. Results and Discussion

### 3.1. Isolation of WCO-Utilizing Strains

After 48 h of static cultivation in the incubator at 37 °C, the purified bacteria were inoculated into the fermentation medium and cultivated in an incubator for 5 days. Three different types of bacteria grew in the fermentation medium, and the results are shown in Table 1. Strain WO2 had the best effect of utilization of WCO, and WO2 grew well in the anaerobic medium. Therefore, the strain WO2 was chosen for follow-up experiments.

**Figure 1:**
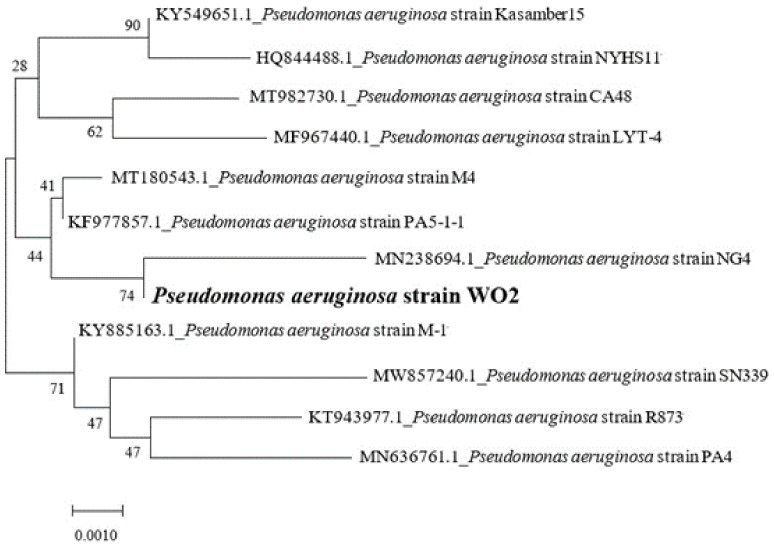
Phylogenetic tree of *Pseudomonas aeruginosa* WO2 based on the Neighbor-Joining method.

The identification results of physiological and biochemical showed that strain WO2 was a facultative, anaerobic, and Gram-negative bacteria. Its growth temperature ranged from 30 °C to 40 °C, the best growth temperature was 32 °C, the acid resistance range was between 5 and 9, and the most suitable bacterial growth pH was 6. The results of the salt tolerance test showed that WO2 could grow well in 6% salinity medium, while the growth of bacteria was seriously inhibited in 8% salinity medium. The 16S rDNA sequence measured by strain WO2 was blast compared in NCBI, and the results showed that more than 99% of the bacteria with homology were *Pseudomonas*. The phylogenetic tree was based on the Neighbor-Joining method (Figure 1). The results show that WO2 and *Pseudomonas aeruginosa* NG4 (Mt982730.1) had the highest nucleotide sequence similarity of 99.62%, so strain WO2 was identified as *Pseudomonas aeruginosa*.

### 3.2. Optimization of WCO Utilization by Pseudomonas aeruginosa WO2

#### 3.2.1. Temperature, Inoculum Ratio, and pH

**Figure 2:**
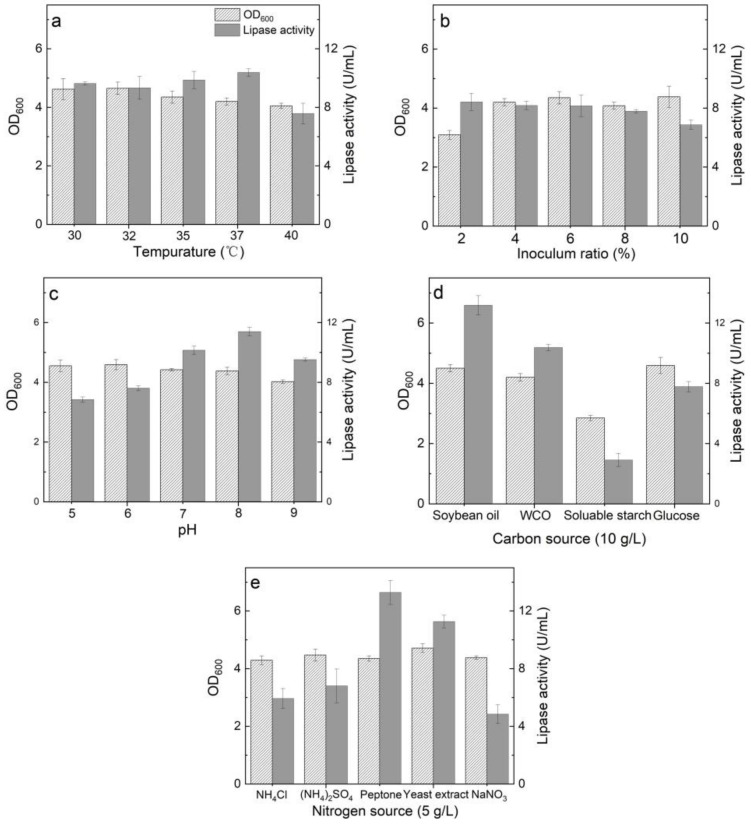
Changes in OD_600_ and lipase activity at various (**a**) temperature, (**b**) inoculum ratio, (**c**) pH, (**d**) carbon sources, and (**e**) nitrogen sources.

Temperature is a crucial parameter affecting the cell growth and function of bacteria. The optimum temperature for cell growth and lipase activity were usually different, so the bacterial growth and lipase activity at different temperatures were determined (Figure 2a). The temperature of the incubator was set to 30–40 °C at 180 rpm for 24 h. *Pseudomonas aeruginosa* WO2 grew well at the selected temperature. Strain WO2 grew best at 32 °C, and the biomass decreased gradually with the increase in temperature. The highest lipase activity of strain WO2 was tested at 37 °C, and the lipase activity decreased to the lowest when the temperature increased to 40 °C.

The influence of inoculum ratio on cell growth and lipase activity of strain WO2 was shown in Figure 2b. Except for 2% (*v/v*) inoculation, cells grew well in other inoculation groups. The optimal inoculum amount was shown to be 6% (*v/v*). In this case, the optical density was the highest and the lipase activity was 8.16 U/mL. Although a 6% (*v/v*) inoculation amount resulted in the highest cell density and lipase activity, the lipase activity decreased in the experimental group with an inoculation amount greater than 6% (*v/v*), which may be because high cell density and rapid growth of bacteria led to the reduction of dissolved oxygen in the serum bottle, thus reducing the utilization of WCO. On the other hand, at 2% (*v/v*) inoculation amount, the growth of bacteria was relatively slow, and the growth period was prolonged, which affected the efficiency of WCO utilization. The bacterial growth and lipase activity of the two experimental groups with 4% (*v/v*) inoculation and 6% (*v/v*) inoculation were similar, so 4% (*v/v*) inoculation was selected for further studies.

pH greatly affected lipase activity (Figure 2c). It was observed that lipase activity was the highest in the medium with pH 8 (11.39 U/mL), consistent with earlier similar results from other studies [37]. In addition, the cells grew well at pH 5~9 with an optimum pH of 7. Previous study has shown that the slightly alkaline environment was conducive to the growth of *Pseudomonas aeruginosa*, but the slightly acidic environment could maximize the conversion of oil into rhamnolipid in the middle and late stage of fermentation [38].

#### 3.2.2. Carbon and Nitrogen Sources

Different carbon sources affect the cell growth and the production of lipase during the fermentation of *Pseudomonas aeruginosa* WO2. In this study, WCO, rapeseed oil, glucose, and soluble starch were selected as a single carbon source. As shown in Figure 2d, the lipase activity was the highest when rapeseed oil was used as the sole carbon source in the medium. When WCO was used as the sole carbon source in the culture medium, the growth of bacteria and lipase activity decreased compared with rapeseed oil. It is speculated that the toxic substances contained in WCO, such as phenylpyridine and aflatoxin, may inhibit the growth of bacteria [30,39]. When glucose was used as a sole carbon source, strain WO2 produced the highest cell biomass, while a significant decrease in lipase activity was observed. In contrast, the worst bacterial biomass and the lowest lipase activity were observed when soluble starch was used as a sole carbon source, with a worse utilization of soluble starch compared with the previous study [35]. Strain WO2 could effectively utilize oil as the ideal substrate and retain more starch matrix when dealing with mixed carbon sources such as FW.

Nitrogen source is an important factor affecting bacterial growth and lipase activity. Two organic nitrogen sources (peptone and yeast extract) and inorganic nitrogen sources ((NH_4_)_2_SO_4_, NH_4_Cl, NaNO_3_) were selected to evaluate the bacterial growth ability and lipase activity of strain WO2 by using WCO as the sole carbon source but different nitrogen sources. As shown in Figure 2e, there was no significant difference in cell density corresponding to multiple nitrogen sources. However, the lipase activity of bacteria was lower in the inorganic nitrogen source experimental group, and the lipase activity of peptone experimental group reached the highest (13.29 U/mL). The increase in lipase activity showed that when organic nitrogen source was used as nitrogen source, the efficiency of consumption of WCO was improved. It should be noted that organic nitrogen source may inhibit the synthesis of rhamnolipid by *Pseudomonas aeruginosa* under aerobic culture conditions [23].

### 3.3. WCO Fermentation Kinetics

**Figure 3:**
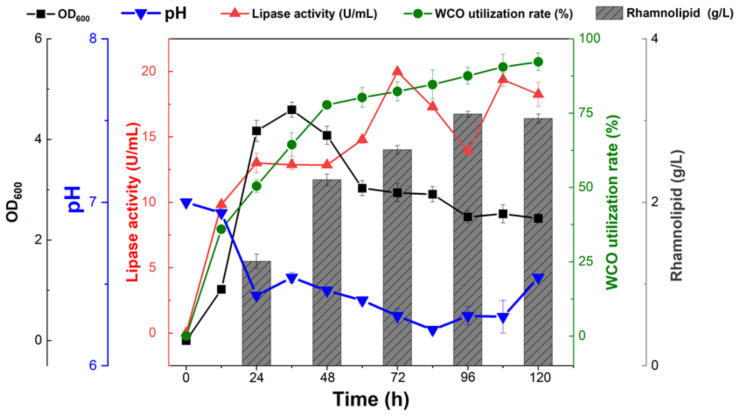
Process of WCO utilization under optimized conditions.

Under the optimum condition for highest lipase activity (37 °C, pH = 7, 4% inoculum ratio, 5 g/L peptone), *Pseudomonas aeruginosa* WO2 grew better and the utilization rate of WCO was improved. The optical density, lipase activity, pH of fermentation medium, WCO utilization rate, and rhamnolipid production are shown in Figure 3. Strain WO2 exhibited a stable phase of bacterial growth at 24 h, and the accumulation of fatty acids and the acidification of fermentation broth had a negative impact on the growth of strain and lipase activity. After cultivation time of 48 h, the decrease in OD_600_ value indicated that the bacterial growth entered the decline phase. With the extension of fermentation time, the pH remained stable, and the oil utilization rate and lipase activity increased. This was consistent with a previous report [31]. The utilization rate of WCO at 48 h is 77.76%. After 5 days of fermentation, the final WCO utilization rate reached 92.25%, and 3.03 g of rhamnolipid was produced. As shown in Table 2, the utilization rate of WCO and the production of rhamnolipid were better than or comparable with results from other studies [33,35,40,41,42].

In addition, during the decline period of the strain, the utilization rate of WCO decreased, only 14.49% WCO was consumed from 48 to 120 h. However, there was a slight decrease in rhamnolipid production in the later stage of fermentation, which indicated that *Pseudomonas aeruginosa* WO2 utilized rhamnolipid. This is consistent with earlier findings that *Pseudomonas* SWP-4 could consume rhamnolipid at the late stage of fermentation [31]. Due to the yield of rhamnolipid decreasing in the later stage of fermentation, 96 h fermentation was the best time for WCO degradation. As shown in Figure 3, the change trend of pH value in the whole fermentation is to decrease first and then increased, reaching the lowest value of 6.22 at 84 h. The main reason for the subsequent increase in pH may be *Pseudomonas aeruginosa* WO2 metabolized rhamnolipid. Furthermore, in order to explore the reason for the best degradation effect after adding organic nitrogen source, the nitrogen source was changed under the optimal degradation conditions.

**Figure 4:**
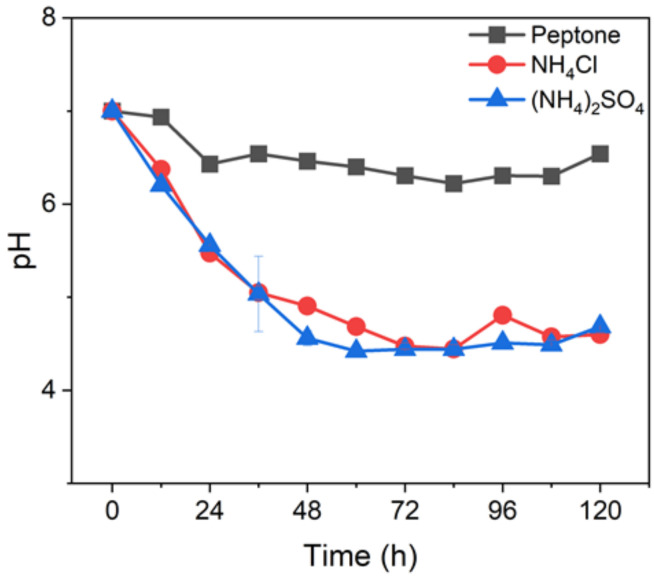
Different nitrogen sources affect the change of pH.

As shown in Figure 4, during the fermentation process, the pH value of the inorganic nitrogen source experimental group decreased significantly. Since the lipase activity of WO2 is the largest at pH = 8, and the amino acid component in the peptone had the ability to buffer pH, the use of an organic nitrogen source stabilized the change of pH and was conducive to WCO utilization. Under the optimal consumption conditions of WCO, the lipase activity of WO2 increased in the late stage of fermentation, which may be due to the consumption of rhamnolipid and to lipase activity increasing with the increase in pH.

**Figure 5:**
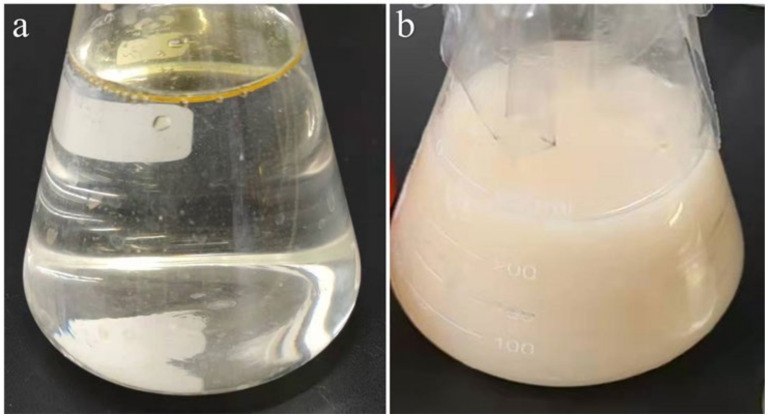
Photograph of WCO fermentation (**a**) initial (0 h) (**b**) After fermentation (120 h).

Interestingly, WCO was observed to float above the medium and its color was yellow under the optimization experiment. WCO was gradually consumed and dispersed into oil droplets. At the same time, the color of the fermentation medium also changed to ivory white. At the end of fermentation, 3.03 g/L of rhamnolipid was produced in the fermentation medium, and no oil droplets were observed in the serum bottle (Figure 5).

### 3.4. WCO Tolerance Test

**Figure 6:**
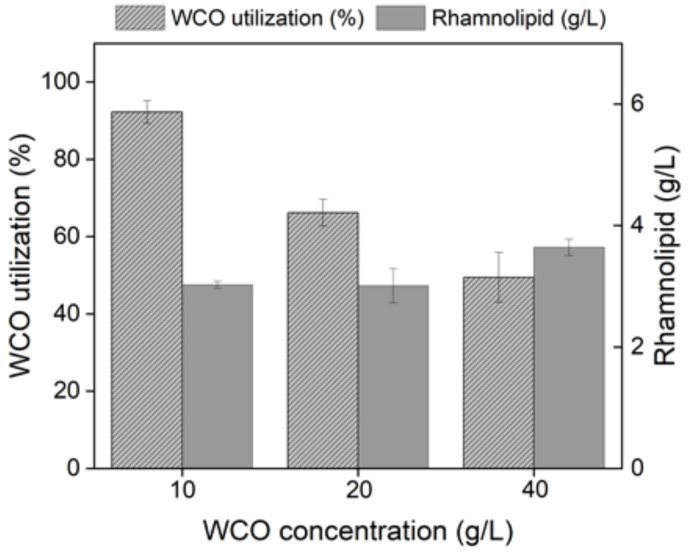
WCO tolerance test of *Pseudomonas aeruginosa* WO2.

Under the optimized conditions, 10 g/L of WCO was consumed 92.25% by *Pseudomonas aeruginosa* WO2, and 3.03 g of rhamnolipid was produced. Increasing WCO content in the medium could increase the effective carbon source, but high WCO concentration caused the oil suspended in the upper layer of the medium. In other words, WCO reduced the gas exchange at the water’s surface and decreased the amount of oxygen in the water. *Pseudomonas aeruginosa* WO2 is facultative anaerobic bacteria, but when there is no nitrate as a nitrogen source, the bacteria carry out aerobic respiration. Therefore, the increase in WCO affected bacterial respiration and growth [43]. As shown in Figure 6, when the initial content of WCO was 10, 20, and 40 g/L, the oil utilization rate was 92.25%, 66.21%, and 49.44%, respectively, and the concentration of rhamnolipid was 3.03, 3.01, and 3.65 g/L, respectively. When the WCO concentration was 40 g/L, the WCO utilization rate reached 49.44%, and nearly 20 g/L WCO was consumed in this experiment. With the increase in WCO, the oil utilization rate decreased gradually, and the production of rhamnolipid increased slightly. Similar phenomena were observed in previous studies [33,35]. Compared with *Pseudomonas aeruginosa* M4 and *Pseudomonas aeruginosa* DR1, increasing substrate concentration could not significantly improve the production of rhamnolipid, and even inhibited the fermentation process. In previous literature, *Pseudomonas aeruginosa* could produce rhamnolipid under aerobic or anaerobic conditions, but the performance and proportion of mono-rhamnolipids congeners were quite different, and adequate oxygen supply increased rhamnolipid production [32,43]. There was a report about high concentration WCO(40 g/L) as a carbon source for rhamnolipid production [31]. It showed that *Pseudomonas* SWP-4 gave a maximum rhamnolipid production around 13.93 g/L. Compared with strain SWP-4, the yield of *Pseudomonas aeruginosa* WO2 was close to it, but strain WO2 was more suitable for low concentration WCO fermentation.

### 3.5. Salt Stress Test

**Figure 7:**
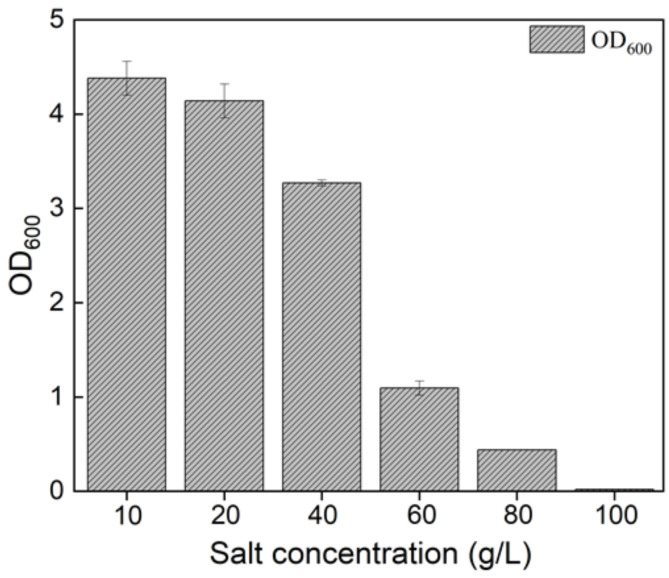
Salt stress test of *Pseudomonas aeruginosa* WO2.

Generally, the salt (NaCl) content in FW is quite high [44], so it was necessary to evaluate the salt tolerance of *Pseudomonas aeruginosa* WO2. The growth of strain WO2 in the medium with different salt concentrations is shown in Figure 7. While the salt concentration was lower than 60 g/L, strain WO2 grew well, and *Pseudomonas aeruginosa* WO2 grew best in the 10 g/L medium. When the salt concentration reached 80 g/L, the bacterial growth was seriously inhibited. According to a previous study [45], the salt content in FW could not seriously inhibit the growth of WO2, which indicates that strain WO2 is an ideal candidate for the utilization of high-salinity oily organic waste or wastewater.

## 4. Conclusions

Bacteria with a high capacity to utilize WCO and produce rhamnolipid may have significant implications for the bioremediation of WCO-contaminated sites. The newly isolated oleophyic *Pseudomonas aeruginosa* WO2 could efficiently utilize WCO to produce rhamnolipid. Oil and peptone were the optimum nutrition sources to maximize lipase activity. Strain WO2 rapidly consumed 77.76% of WCO after 48 h and produced rhamnolipid 3.03 g/L at the end of fermentation. The oil utilization rate, rhamnolipid concentration, and productivity were 87.56%, 3.08 g/L, and 0.032 g/L·h at 96 h, respectively. Moreover, strain WO2 possessed superior WCO utilization and salt tolerance to exhibit much higher resistance to the noxious environmental conditions. These unique characteristics demonstrate the potential of *Pseudomonas aeruginosa* WO2 for the utilization of WCO to bioenergy production without addition of lipid enzymes. Overall, *Pseudomonas aeruginosa* WO2 presented interesting features. More importantly, the oily sludge may provide new strains with biotechnological potential, and open perspectives for WCO energy conversion.

## Figures and Tables

**Table 1:** WCO utilization characteristics of the three separated strains.

Strain Number	WO1	WO2	WO3
Bacterial colony color	Vermilion	Chartreuse	Ivory white
WCO utilization rate (%)	62.03 ± 3.29	86.50 ± 0.85	37.53 ± 3.71
Lipase activity (U/mL)	8.426 ± 0.350	8.155 ± 0.532	5.093 ± 1.344

**Table 2:** Performance of different organism during waste oil-based rhamnolipid fermentation.

Organism	Carbon source	Rhamnolipid (g/L)	Rhamnolipid Yield (g/g)	References
*Pseudomonas aeruginosa* DR1	Mango kernel oil (1%)	1.80	0.18	[33]
*Pseudomonas aeruginosa* M4	Waste cooking oil (2.5%)	1.12	0.045	[35]
*Pseudomonas aeruginosa* strain B	Kitchen waste oil (2%)	2.47	0.123	[40]
*Pseudomonas aeruginosa* ATCC 9027	Petroleum oil waste (2%)	2.70	0.135	[41]
*Pseudomonas aeruginosa* PAO1	Olive mill waste (0.2%)	0.30	0.15	[42]
*Pseudomonas aeruginosa* WO2	Waste cooking oil (1%)	3.03	0.328	This study

## Data Availability

Not applicable.
